# Spectral data of nicotabaflavonoidglycoside

**DOI:** 10.1016/j.dib.2018.06.081

**Published:** 2018-06-26

**Authors:** Cai-Yan Yang, Yao Lin, Hui-Xiong Yuan, Wen-Pei Yang, Xian Wei, Zu-Liang Huang

**Affiliations:** a The Pharmaceutical School of Youjiang Medical University for Nationalities, Baise 533000,China; b The Key Laboratory of Guangxi Colleges and Universities in Youjiang River Drainage Area for Studying Traditional Chinese Medicinal Herbs (folk medicine), Baise, 533000 China; c The Affiliated Hospital of Youjiang Medical University for Nationalities, Baise 533000, China

## Abstract

The dataset addressed in this article relates to the research article entitled“Nicotabaflavonoidglycoside, the first example of cembranoid and flavonoid heterodimer from *Nicotiana tabacum*” (Yang et al., 2018) [1]. The dataset presents the MS_4_(879-571-421-335), MS*^n^* fragment pathways, (+) HR-ESI-MS, (−) HR-ESI-MS, UV, IR, ^1^H NMR, ^13^C NMR, HSQC, ^1^H–^1^H COSY, HMBC, ROESY, ORD and ECD data of Nicotabaflavonoidglycoside. The MS_4_(879-571-421-335), (+) HR-ESI-MS, (−) HR-ESI-MS, UV, IR, ^1^H NMR, ^13^C NMR, HSQC, ^1^H–^1^H COSY, HMBC, ROESY, ORD and ECD data were collected by experimental methods, and the MS^n^ fragment pathways were acquired by analyses.

**Specifications Table**

**Table:** 

Subject area	*chemistry*
More specific subject area	*Natural Medicine Chemistry*
Type of data	*Table, text file, Scheme*
How data was acquired	*NMR, mass spectrometry, IR,UV,ECD, ORD; LCMS-IT-TOF mass spectrometer, Advance III-600 NMR spectrometers, Bio-Rad FTS-135 spectrometer, UV-2401A spectrophotometer, Applied Photophysics Chirascan instrument, Jasco model 1020 polarimeter.*
Data format	*Raw, analyzed, etc.*
Experimental factors	*Sample was dissolved by acetone-d*_*6*_*or MeOH*
Experimental features	*Instrumental test*
Data source location	*Baise, China*
Data accessibility	*data is with this article*
Related research article	*Nicotabaflavonoidglycoside, the first example of cembranoid and flavonoid heterodimer from Nicotiana tabacum*

**Value of the data**

• This data were compared with the data of cembranoids and flavonoids for further insight to research heterodimer.
• This data can serve as a benchmark for other researchers to elucidate the structures of cembranoids and flavonoids.
• The data were used in the development of further experiments in the area of natural medicine chemistry.

## 1 Data

**Scheme 1:**
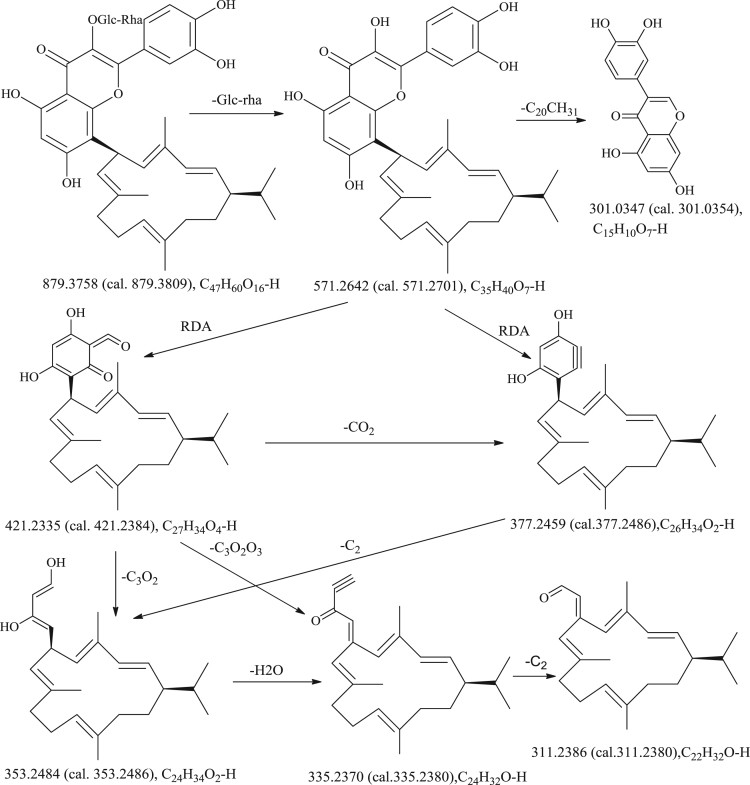
The MS^n^ fragment pathways.

[*α*]_24 D_: +24.5 (c 0.20, MeOH); UV (MeOH) *λ*_max_ (log *ε*) 249 (4.40), 375 (3.90) nm; ECD (CH_3_OH) ∆*ε* 196+11.14, ∆*ε* 208−5.00, ∆*ε* 214 − 2.84, ∆*ε* 226−9.28, ∆*ε* 250+7.97, ∆*ε* 257+7.18, ∆*ε* 264+8.41, ∆*ε* 266+8.47, ∆*ε* 306−1.24; IR (KBr) *ν*_max_ 3442, 1648, 1608, 1511, 1442, 1384, 1357, 1303, 1272, 1205, 1160, 1066 cm^−1^; (−) HRESIMS *m/z* 879.3765 [M−H]^−^ (C_47_H_59_O_16_ calcd 879.3809), 903.3719 [M+Na]^+^ (C_47_H_60_O_16_Na calcd 903.3774), 881.3896 [M+H]^+^ (C_47_H_61_O_16_ calcd 881.3954) ( Tables 1, 2 and Scheme 1).

**Table 1:** ^1^H (600 MHz) and ^13^C (150 MHz) NMR Data in acetone-*d*_6_ (*δ* in ppm, *J* in Hz).

No.	*δ*_H_	*δ*_C_	No.	*δ*_H_	*δ*_C_	No.	*δ*_H_	*δ*_C_
Rutin moiety								
2		158.8, s	1′		123.0, s	4′″	3.35, m	74.0, d
3		135.8, s	2′	8.05, s	118.7, d	5′″	3.37, m	76.9, d
4		179.5, s	3′		145.7, s	6′″	3.48, m	67.8, t
							3.72, dd, 17.9, 6.8	
5		160.8, s	4′		149.4, s	1″″	4.60, brs	102.0, d
6	6.38, s	100.2, d	5′	7.06, d, 8.2	116.2, d	2″″	3.66, d, 5.5	71.8, d
7		162.8, s	6′	7.78, d, 8.2	122.5, d	3″″	3.46, m	70.4, d
8		110.3, s	1′″	5.16, d, 7.5	105.7, d	4″″	3.66, m	72.1, d
9		154.5, s	2′″	3.47, m	75.5, d	5″″	3.47, m	68.9, d
10		105.7, s	3′″	3.47, m	78.4, d	6″″	1.12, d, 5.5	18.1, q
5-OH 12.44, s								
Diterpene moiety (**DM**)								
1″	1.88, m	49.6, d	8″		133.0, s	15″	1.47, m	33.4, d
2″	5.31, dd, 15.2, 10.0	132.7, d	9″	a 2.94, t, 11.8	31.4, t	16″	0.76, d, 6.3	20.8, q
				b 1.86, m				
3″	6.83, d, 15.2	131.0, d	10″	a 2.40, m	24.1, t	17″	0.86, d, 6.3	20.9, q
				b 2.04, m				
4″		133.0, s	11″	5.06, d, 10.0	125.6, d	18″	1.80, s	21.2, q
5″	6.08, d, 10.2	128.6, d	12″		131.7, s	19″	1.62, s	22.5, q
6″	5.79, t, 10.2	33.0, d	13″	2.02, m	37.6, t	20″	1.66, s	15.0, q
7″	6.08, d, 10.2	129.1, d	14″	a 1.86, m	28.6, t			
				b 1.19, m

**Table 2:** Accurate masses and elemental compositions from negative ESI-IT-TOF MS^n^ experiments.

MS^n^	Precursor ion	Product ion	Calculated *m*/*z*	Measured *m*/*z*	Error *m*/*z*	Error ppm	Elemental composition
MS^1^	compound **1**	879	879.3809	879.3758	−5.1	−5.80	C_47_H_60_O_16_
MS^2^	879	879	879.3809	879.3754	−5.5	−6.25	C_47_H_60_O_16_
		571	571.2701	571.2642	−5.9	−10.33	C_35_H_40_ O_7_
MS^3^	879-571	421	421.2384	421.2335	−4.6	−11.63	C_27_H_34_O_4_
		377	377.2486	377.2460	−2.6	−6.89	C_26_H_34_O_2_
		353	353.2486	353.2464	−2.2	−6.23	C_24_H_34_O_2_
		335	335.2380	335.2370	−1.0	−2.98	C_24_H_32_O
		301	301.0354	301.0347	−0.7	−2.33	C_15_H_10_O_7_
MS^4^	879-571-421	377	377.2486	377.2459	−2.7	−7.16	C_26_H_34_O_2_
		353	353.2486	353.2486	−2.2	−6.23	C_24_H_34_O_2_
		335	335.2370	335.2380	−1.0	−2.98	C_24_H_32_O
		311	311.2380	311.2386	0.6	9.00	C_22_H_32_O

## 2 Experimental design, materials, and methods

Nicotabaflavonoidglycoside was obtained by various chromatography methods. The optical rotation, HRESIMS data, UV spectra, Electronic circular dichroism (ECD) spectra and IR of nicotabaflavonoidglycoside were recorded on Jasco model 1020 polarimeter, LCMS-IT-TOF mass spectrometer, UV-2401A spectrophotometer, Applied Photophysics Chirascan instrument and Bio-Rad FTS-135 spectrometer, respectively. Its 1D and 2D NMR spectra were acquired using Advance III-600 NMR spectrometers (Bruker, Bremerhaven, Germany) with TMS as internal standard.
