# Supplementary material for: Spectral data of nicotabaflavonoidglycoside
Source: Data Brief. 2018 Jun 26;19:1545–8. doi: 10.1016/j.dib.2018.06.081 (PMC6141958; doi:10.1016/j.dib.2018.06.081)
Supplement: Supplementary file 1 — Supplementary material [file mmc1.docx]

Declaration of interest

The authors declare no conflicts of interest among all authors in the manuscript of “Nicotabaflavonoidglycoside, the first example of cembranoid and flavonoid heterodimer from *Nicotiana tabacum*.”and “Spectral Data of Nicotabaflavonoidglycoside**”**

Declarer: Cai-Yan Yang, Yao Lin, Hui-Xiong Yuan, Wen-Pei Yang, Xian Wei, Zu-Liang Huang

May. 23^th^, 2018
